# Clinicopathological Characteristics of Acute Antibody-Mediated Rejection in Pediatric Liver Transplantation—A Single-Center Study

**DOI:** 10.3390/jcm15093554

**Published:** 2026-05-06

**Authors:** Sylwia Szymanska, Barbara Piątosa, Mateusz Ciopiński, Artur Kijewski, Piotr Kaliciński, Małgorzata Markiewicz-Kijewska

**Affiliations:** 1 Department of Pathology, Children’s Memorial Health Institute, Al. Dzieci Polskich 20, 04-730 Warsaw, Poland; 2 Histocompatibility Laboratory, Children’s Memorial Health Institute, 04-730 Warsaw, Poland; b.piatosa@ipczd.pl; 3 Department of Pediatric Surgery and Organ Transplantation, Children’s Memorial Health Institute, 04-730 Warsaw, Poland; m.ciopinski@ipczd.pl (M.C.); p.kalicinski@ipczd.pl (P.K.); 4 Student Scientific Club “Kasai”, Department of Pediatric Surgery and Organ Transplantation, Children’s Memorial Health Institute, 04-730 Warsaw, Poland; kijewski.a@wp.pl

**Keywords:** liver transplantation, rejection, antibody-mediated rejection, C4d

## Abstract

**Introduction:** Presently, liver transplantation is becoming a more common treatment option for adults and children suffering from liver failure. Antibody-mediated rejection (AMR), a phenomenon that is exceedingly uncommon and inadequately comprehended, may induce graft dysfunction. The objective of the investigation was to evaluate the clinical and histopathological manifestations of AMR in pediatric patients. **Material and methods:** The retrospective study comprised sixty-two liver core biopsies from forty-two pediatric patients. In a total of 10 biopsies, 7 children were diagnosed with AMR, while 35 of them exhibited features of acute T-cell-mediated rejection (TCMR) in 52 biopsies. The C4d binding assay was conducted in all biopsies using the immunohistochemical (IHC) method. Bilirubinostasis, steatosis and acute and chronic rejection were re-assessed in all specimens. The 6-grade Ishak scale was employed to evaluate fibrosis. The TCMR activity was established using the Banff classification. AMR was assessed according to a novel histopathological grading system that was developed by the authors. Depending on the type of rejection, the relationship between histopathological grading, morphological characteristics, and laboratory parameters was established for each group. Standard methods were implemented to conduct statistical analysis. **Results:** At the time of biopsy, the median age of patients was 47.6 months (15.03–98.83) and the median time from transplantation was 0.9 months (0.3–7.6). The study’s findings provided evidence that histopathological lesions were the least specific manifestation, which supported the presence of AMR. A positive C4d staining statistically increases the likelihood of AMR diagnosis, whether or not there are associated morphological abnormalities. The type of rejection and laboratory tests did not exhibit any statistically significant correlation. **Conclusions:** The diagnosis of AMR in a transplanted liver is intricate and requires a multifaceted approach. However, the proposed histopathological grading may be a helpful method for selecting patients who should be assessed for donor-specific antibodies (DSAs) or in whom AMR should be suspected when DSAs cannot be determined.

## 1. Introduction

At present liver transplantation (Ltx) is the preferred treatment for end-stage organ failure. Quality of life following transplantation, particularly in the initial stages of recovery, is comparable to that of the general population [1]. Regrettably, the quantity of potential recipients continues to surpass the quantity of potential donors. It is imperative to preserve graft function for an extended period in order to achieve equilibrium between the availability of organs for transplantation and the needs of the body. Rejection is a prevalent cause of organ dysfunction, with T-cell-mediated (TCMR) being the most common [2]. In order to diagnose this form of rejection, core needle biopsy is necessary, as is the administration of steroid pulses [3]. The likelihood of success is high, provided that no steroid-resistant rejection develops. Diagnostically and therapeutically, antibody-mediated rejection (AMR) presents a challenge. For a long time, the liver has been regarded as an immunologically privileged organ that is not impacted by immunologically induced damage due to its size, good vascularization, the facilitation of phagocytosis by Kupffer cells, regenerative capacity, and the secretion of soluble human leukocyte antigen (HLA) that can bind to and opsonize harmful antibodies and immune complexes [4]. The transplanted liver can be damaged by immunological processes as is the case with other solid organs. This is currently recognized.

The Banff criteria, which were initially established in 2016 [5] and were most recently updated in 2021 [6], necessitate the co-occurrence of clinical symptoms (most frequently fever, jaundice and fatigue), the presence of histopathological changes in the biopsy, a positive C4d reaction (performed by immunohistochemical method), and the presence of donor-specific antibodies (DSAs). Immunosuppression modification, plasmapheresis and IVIG are the primary treatment options, although protocols are still in the process of being developed. In cases of severe unresponsiveness, retransplantation may be considered. Regrettably, the histopathological lesions in AMR are less well-defined than those in TCMR. The most indicative feature of this process is the presence of C4d deposits in microvessels. It is, however, feasible that AMR may manifest in the absence of C4d deposits, as is the case of renal transplantation [7,8]. A potential explanation for this phenomenon was sought in mechanisms that are not dependent on HLA DSAs, the involvement of non-HLA allo- or autoantibodies, or antibody-independent NK cell activation [9]. Moreover, it is necessary to consider the time-dependent factor, as the reactions may be negative due to insufficient antibody generation (below the detection limit), insufficient antigen–antibody binding that is unable to induce complement binding, or too late (disconnection has already occurred as in the case of endothelial regeneration or in the late phases of the process). Liver transplantation may potentially involve comparable mechanisms. Therefore, the objective of this study was to determine whether the criteria derived from the model described in heart transplantation in 2013 [10], in conjunction with microscopic and/or immunohistochemical features in the context of clinical data, could be employed as a simple, repeatable grading method for the histopathological assessment of AMR in livers transplanted in children and the identification of patients at a heightened risk of developing AMR or undergoing treatment-resistant rejection.

## 2. Material and Methods

In the retrospective study conducted at a single center between 2005 and 2022, sixty-two archival core needle biopsies from forty-two pediatric patients after liver transplantation were included. All biopsies were performed for clinical reasons (deterioration of graft function and suspicion of rejection). The Children’s Memorial Health Institute Bioethics Committee authorized the investigation (approval no 11/KBE/2023) on 15 March 2023. The study was conducted in accordance with the Helsinki Declaration. The investigation enrolled biopsy specimens from 2013 to 2022 and the analysis was performed between 2023 and 2025. The study was conducted with the written consent of the legal guardians of all patients and children aged 16 and older. The datasets presented in the study are unavailable due to patients’ privacy.

Utilizing the manufacturer’s instructions, the Luminex single antigen bead assay was implemented to assess anti-HLA antibodies. Briefly, antigen-coated beads are incubated with patient’s serum. Antibodies against individual antigens bind to specific antigens. The complex is detected by PE-conjugated anti-human IgG, which binds to the antigen–antibody complex on beads. Unbound antibodies are washed out. Fluorescence emitted by the complex generated by a PE-conjugated anti-human antibody bound to an antibody from a patient’s serum is measured by a Luminex instrument and analyzed using a commercial dedicated software. The result considered positive was MFI > 1000.

The sera that were tested were obtained at the time of biopsy or within a reasonable time frame, with a maximum of seven days.

The biopsies were re-evaluated by two independent pathologists who were not aware of the clinical data. The following criteria were considered for inclusion: a sufficient quantity of tissue material to conduct potential additional immunohistochemical tests (IHC), complete clinical data, and anti-HLA antibody determination at the same time or shortly after the biopsy, with a maximum of 7 days between the two. Incomplete clinical data, non-diagnostic biopsies, and the absence of anti-HLA evaluation were regarded as exclusion criteria. The presence of bilirubinostasis, steatosis and fibrosis (6-point Ishak scale) as well as TCMR (assessed according to the Banff criteria) was verified using slides that were routinely stained with hematoxylin and eosin (HE) and analyzed in light microscopy.

A histopathological AMR grading scale (pAMR) was employed to describe the staining results, which was modeled after the scale that was adopted by the International Society for Heart and Lung Transplantation (ISHLT) in 2013 for hearts transplanted:

- 1H (+) histopathological changes that are indicative of AMR are present, and C4d reaction is negative.
- 1I (+) C4d reaction is positive despite the absence of histological changes.
- 2—the histopathological lesions are present and IHC reaction is positive.

When at least two of the following features were present, category 1h+ was diagnosed:

- Prominent portal capillaritis, endothelial cell swelling, and dilated capillary spaces;
- Microvasculitis (neutrophils and eosinophils within vessels);
- Portal/peri-portal edema;
- Centrilobular/perivenular hepatocyte swelling.

IHC staining samples were fixed in 4% phosphate-buffered formalin and after 24 h stored in 70% ethanol (POCH S.A., Gliwice, Poland) until further processing. Tissues were then dehydrated and paraffin embedded, according to standard histological techniques. The paraffin blocks were cut in slices (5 µm), which were mounted on silanized microscope slides (Sigma-Aldrich, Hamburg, Germany). Then, the slides were de-waxed in xylene and were hydrated in a graded series of ethanol to PBS. Antigen retrieval slices were heated in a microwave (650 W) in 400 mL of 10 mM citrate buffer (pH 6.0), twice for 5 min with a 5 min pause. After 30 min of cooling, the slices were rinsed with PBS (Sigma-Aldrich, Hamburg, Germany). A standard protocol of staining was then applied, using EnVision System HRP (DAB) (DAKO, Glostrup, Denmark) with specific polyclonal antibodies against C4d (Biomedica Grouppe, C4d, DAKO, Biomedicia, Denmark, dilution 1:40, C4d). A positive reaction was determined in accordance with the Banff recommendations, when linear, non-granular staining was observed in portal veins and capillaries, as well as in the endothelium of sinusoidal and central veins. The grade was classified as negative (score 0), minimal (<10%, score 1), focal (10–50%, score 2), or diffuse (score 3).

In order to identify fibrous connective tissue, histochemical Masson’s trichrome staining was implemented in all biopsies.

During the final phase of the project, the clinical condition of patients was evaluated in relation to the changes observed in biopsies. This included initial condition, the treatment used, its effectiveness, and the potential correlation with the severity of histopathological changes.

Frequencies and percentages employed to represent categorical variables in statistical continuous variables were tested for normality using the Shapiro–Wilk test, and due to non-normal distribution are presented as medians with interquartile ranges (IQRs). Comparisons of continuous variables between two independent groups were performed using the Mann–Whitney U test. Categorical variables were compared using Fisher’s. A *p*-value < 0.05 was considered statistically significant. Analyses were performed using Statistica 13.3 (StatSoft Inc., Tulsa, OK, USA).

## 3. Results

A total of 10 (16%) of the 62 archival core needle biopsies from seven pediatric patients (pts) (17%) were diagnosed with AMR, while 52 (84%) of the biopsies from 35 pts (83%) were diagnosed with TCMR with varying degrees of severity. The study comprised forty-two patients (20 girls and 22 boys) with a median age of 47.6 months (15.03–98.83) at the time of biopsy and a median time from transplantation of 0.9 months (0.3–7.6). Indications for transplantation included biliary cirrhosis (20 pts, covering 17 due to biliary atresia, 2 due to Progressive Familial Intrahepatic Cholestasis/PFIC/ and 1 iatrogenic case), autoimmunological disorders (e.g., AIH, PSC-5 pts), acute liver failure (5 pts), cryptogenic cirrhosis (3 pts), non-resective liver tumors (Hepatocellular carcinoma/HCC/, Hepatoblastoma/HBL/-2 pts), Budd–Chiari syndrome (1 pt), Alpha 1 antitrypsin deficiency (1) and the need for retransplantation (ReLtx-4 pts). In 28 (67%) cases, elective surgery was performed, while 2 (4%) cases were semi-elective (tumors). In four (9%) cases, the surgery was acute on chronic mode and in eight (20%) it was urgent. In total, 11 (26%) liver patients underwent ABO incompatible transplantation, with 5 patients exhibiting AMR and 6 children exhibiting TCMR. The clinical characteristics of both groups are comprehensively compared in Table 1.

In 20 pts, primary immunosuppression consisted of two drugs (tacrolimus and mycophenolate mofetil or glucocorticosteroids), while in 22 pts, a triple immunosuppression protocol (tacrolimus, mycophenolate mofetil, and glucocorticosteroids) was implemented. The induction treatment was administered to 10 pts with basiliximab and 1 pt with rituximab.

Regarding pAMR, a statistically significant difference was demonstrated between the groups for categories 1(I+) (*p* < 0.001) and 2 (*p* < 0.01). This indicates that histopathological lesions (*p* = 0.577) were the least specific manifestation suggesting AMR. The probability of diagnosing AMR is statistically increased by a positive C4d staining result, regardless of whether or not it is accompanied by morphological abnormalities.

In 9 out of 10 biopsies with AMR, microvasculitis was the most prevalent histopathological lesion, and it was the sole change indicative of AMR in six cases. Portal capillaritis was identified in three biopsies, with two exhibiting minimal intensity, and one being the most prominent. Dilation of capillaries was observed in 2 biopsies, while centrilobular/perivenular hepatocyte swelling was present in 4. One biopsy revealed endothelial cell swelling. Three biopsies were diagnosed with C4d score 3, three more with score 2, and four with score 1. Class II-specific anti-HLA antibodies were detected in six patients, and class I in one patient. The characteristics of patients with AMR are comprehensively present in Table 2.

**Figure 1:**
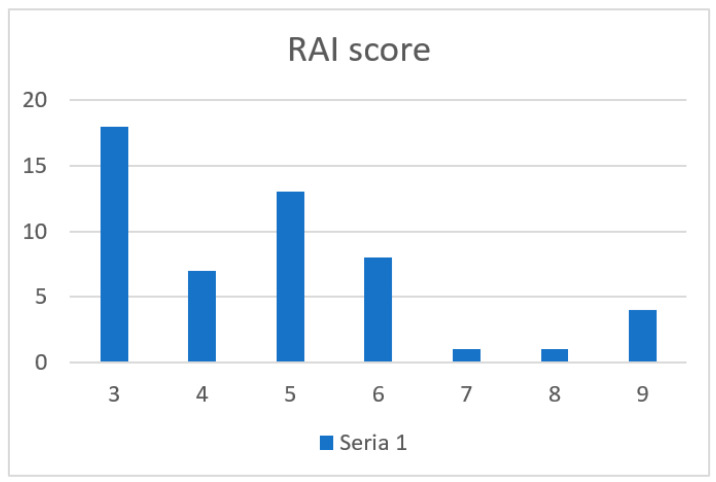
The rejection activity index (RAI) in individual TCMR cases.

Neither of the patients in the TCMR group exhibited any characteristics that Banff deemed indicative of AMR. Portal inflammation, bile duct damage, and venous endothelial inflammation of varying degrees were present in all biopsies. The diagram (Figure 1) is provided to illustrate the rejection activity index (RAI) in individual TCMR cases.

No statistically significant correlation was observed between the type of rejection and laboratory tests (Table 3) as well as advanced fibrosis (Ishak score 4–6) (*p* = 0.185). Nevertheless, a minimal increase in fibrous connective tissue (Ishak score 1–3) was statistically more prevalent in biopsies with TCMR (*p* = 0.026). Steatosis was more prevalent in patients with AMR (*p* = 0.030), despite the fact that no difference was observed in the presence of bilirubinostasis (*p* = 0.291).

## 4. Discussion

**Figure 2:**
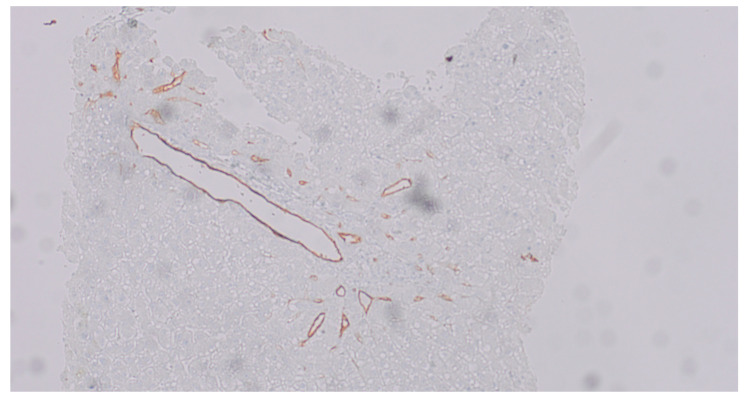
Diffuse (score 3) positive C4d linear, non-granular staining in portal veins and capillaries, IHC, magnification ×10.

**Figure 3:**
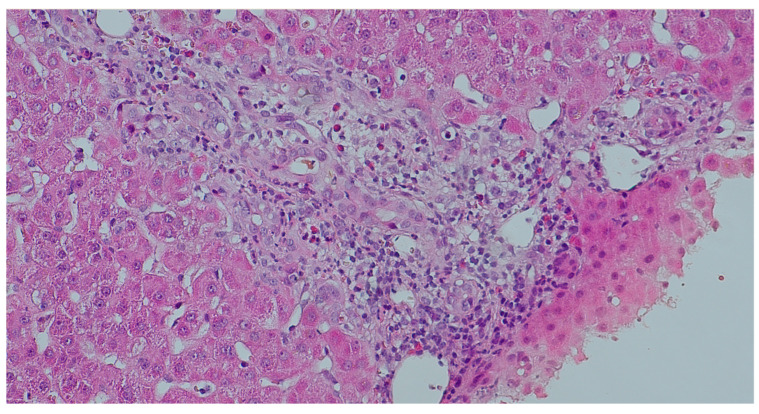
Portal space with the presence of mixed inflammatory infiltrates with eosinophilia, vein endothelial cell hypertrophy and venulitis, H&E, magnification ×10.

**Figure 4:**
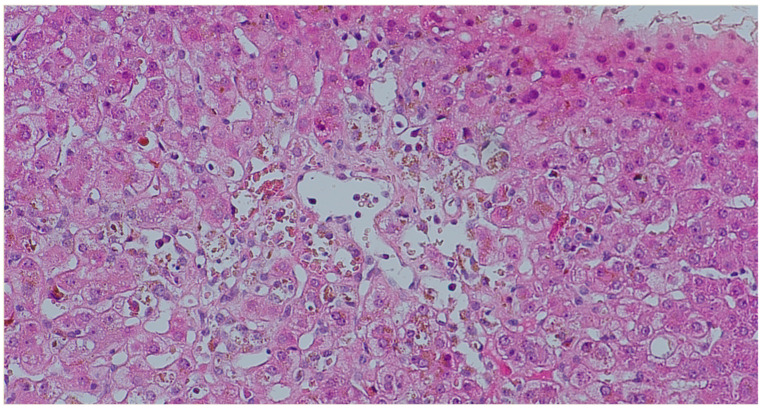
Sinus vessels with features of venulitis and hepatocytes with bilirubinostasis, H&E, magnification ×10.

Liver transplant rejection occurs when the recipient’s immune system perceives the new organ as foreign and attacks it. The first few months are the most common time for TCMR to occur, which is a form of rejection concerning 15–25% of cases. It is typically manageable through the use of heightened steroids or modifications to immunosuppression. AMR, on the other hand, is a substantial cause of graft damage and is the result of recipient’s immune system producing antibodies that attack the new organ, targeting specific donor antigens /HLA/. However, its inherent tolerogenic nature results in its underrecognition in the liver. AMR of the liver is diagnosed through clinical signs, DSAs, C4d staining, and biopsy findings. The treatment is more aggressive than in the case of TCMR, which is why it is essential to make a rapid and accurate diagnosis. The kidney has been the site of AMR cases, particularly chronic ones, in which the C4d reaction was negative [11,12,13]. It appears that the liver may exhibit a similar scenario, particularly in light of the lack of clarity regarding the clinical course of AMR in relation to pathologic findings and treatment, as well as its association with DSA. Therefore, the primary objectives of the study was to characterize this phenomenon in the pediatric population, with the particular emphasis on morphological lesions. A novel scale for the histopathological evaluation of AMR was developed for this purpose, based on the one that was implemented in 2013 for the diagnosis of transplanted hearts [14]. The presumption was that the grading should be easy to use and should enable the identification of patients for whom a DSA determination is required. The scale assessed the presence of histopathological changes that are indicative of AMR and/or positive C4d expression. The statistical analysis revealed that histopathological changes were the least specific feature of AMR, and they are significantly less specific than those in TCMR. The probability of AMR was elevated when the C4d reaction was positive (p1I or p2). Figure 2 presents a diffuse (score 3) positive C4d linear, non-granular staining in portal veins and capillaries. Nonetheless, it is crucial to consider that the reaction may have been performed prematurely, resulting in the antibody not having the opportunity to bind to the antigen, or it may have been performed too late, causing the antibody to have already separated (transition to chronic phase). Therefore, the DSAs marking is essential and their presence at high level (often indicated by high Mean Fluorescence Intensity—MFI > 1000) [13] confirms the diagnosis of AMR. Numerous endeavors have been undertaken to establish a histopathological index for the assessment of AMR in transplanted liver [14,15]. O’Leary et al. [14] demonstrated that portal eosinophilia, portal vein endothelial cell hypertrophy and eosinophilic central venulitis were significantly associated with AMR. Eosinophilia was not a permanent phenomenon in our group, despite the fact that it was visible in the portion of the biopsy. Mixed inflammatory infiltrates with eosinophilia, vein endothelial cell hypertrophy and venulitis within portal space, which are present in Figure 3. Microvasculitis was the most frequently observed lesion (Figure 4). Cicalease et al. [15] employed a semi-quantitative grading system to characterize histopathological features that are indicative of AMR, a method that is exceedingly precise, but which is excessively complex for practical applications. Adult patients were the subjects of both studies. Although the presence of at least two of the changes included in the Banff criteria was required to establish the H1(+) category, histopathological lesions without positive C4d reaction did not correlate with diagnosis of AMR in our study. This outcomes underscores the validity of the 2016 recommendations [5], which emphasize the necessity of a positive C4d reaction and high DSA titers in addition to tissue damage to establish AMR. Furthermore, the analysis was conducted on a pediatric group. The result may also be influenced by the fact that the changes are frequently more subtle in children than in adults. The 2016 Banff classification [7], while introducing AMR as an official category in liver transplantation, also proposed a grading of histopathological changes (h-score). The h-score assesses the severity of microvascular changes, and values of 2 and 3 are considered to be indicative of AMR. The system proposed in our study largely followed the Banff recommendations but was enriched by taking into account the c4d reaction.

Microvasculitis and marked capillary dilation were the most prevalent histopathological lesion in our group. Prominent endothelial cell enlargement, portal edema, and fibrin deposition/red cell extravasation were not that common. However, inflammation accompanied by mild eosinophilia was observed frequently. These changes were significantly associated with AMR in O’Leary’s study. Our original, easy-to-use system combines the h-score and C4d-score introduced by Banff in 2016 [7].

In this study, we also investigated whether there is a higher incidence of laboratory parameter disturbances that reflect liver function during specific types of rejection. However, any statistically significant relationship was not proved. Additionally, an effort was made to evaluate the correlation between rejection and various histopathological lesions. The results indicated that there was no correlation between bilirubinostasis and TCMR or AMR. Cholestasis is identified as a feature indicative of AMR, however, this pertains to chronic cases that were not incorporated into this study. Moreover, bilirubin retention in the acute phase is more frequently a result of rejection which causes damage to the bile ducts.

Conversely, steatosis was statistically more prevalent in AMR biopsies than in TCMR biopsies in our group. The literature indicates that a higher risk of TCMR and bile duct loss (indicative of chronic ductopenic rejection) is associated with donor liver macrovesicular steatosis—specifically steatosis ≥ 30% [16]. The relationship between steatosis and rejection has not been the subject of any research to date. Steatosis is a multifactorial process, which may be associated with recipient obesity, metabolic syndrome, or Hepatitis C subsequent to transplantation. It also indicates an increased risk of fibrosis progression. In our cohort, patients diagnosed with AMR had a higher weight than those diagnosed with TCMR, despite their similar ages. Furthermore, steroids were administered to all but one of the children with AMR, whereas 22 of the 35 patients in TVMR group received steroids. The development of steatosis may have been influenced by these factors.

Fibrosis and chronic damage are significant challenge in the field of contemporary liver transplantation. In children, particularly adolescents, noncompliance and the subsequent development of ductopenia may occur. We therefore investigated whether the severity of fibrosis, as indicated by precise 6-point Ishak scale, is more prevalent in patients who has experienced rejection. The association between any of the forms of rejection and advanced fibrosis (Ishak score 4–6) was not established. Nevertheless, biopsies with TCMR exhibited a statistically more frequent slight increase in fibrous connective tissue (Ishak score 1–3). Various factors, such as the number of rejection episodes and the time following transplantation, contribute to the progressive fibrosis of the organ. Wesley et al. [17] proved that allograft fibrosis was prevalent; however, it was principally mild and relatively stable for 5 (and 10) following the transplant. In the study, 74% of the biopsies exhibited minimal fibrosis at 6 months post-transplant. The mean time since transplantation in our group was 0.9 months. It is a relatively early stage following the procedure, which precludes the development of advanced fibrosis. The reason for the absence of advanced fibrosis at the time of biopsy may be due to a previous episode of rejection or inflammation, which may have contributed to the increase in fibrous connective tissue. Although it is challenging to ascertain the reason for the increased frequency of fibrous connective tissue in TCMR biopsies, it might be related to previous changes in the donor.

The study concentrated on AMR cases; therefore, the impact of this diagnosis on the graft’s continued functionality was examined.

One of the patients with AMR underwent three consecutive biopsies within a month. A positive C4d reaction with a score 3 persisted each time, despite the fact that histopathological changes subsided. Plasmapheresis, methylprednisolone, and intravenous immunoglobulins (IVIG) were administered to the patient. He eventually experienced an improvement in his clinical condition, which is currently stable. No evidence of rejection or substantial fibrosis was observed in the most recent protocol biopsy.

Another patient exhibited signs of AMR in two consecutive biopsies that were conducted 12 weeks apart. He was administered IVIG and subsequently rituximab. The subsequent follow-up biopsy did not report rejection, and the last protocol biopsy did not reveal any significant abnormalities.

The remaining children with AMR exhibited changes in only one biopsy and their condition improved following treatment (antithymocyte globulin /ATG/, in some cases in conjunction with plasmapheresis). Post-transplant lymphoproliferative disease (PTLD) in the form of Classical Hodgkin’s Lymphoma was diagnosed in one of these children four years after an episode of rejection. Minor nonspecific inflammatory infiltrates or minimal steatosis were the only abnormalities observed in subsequent biopsies of the remaining patients.

These observations indicate that allograft loss is not always a consequence of AMR, when it is promptly identified and aggressively treated; however, the repercussions can occasionally be severe. Nevertheless, our experience, comparable to that of other centers, is restricted by the dearth of patients.

The retrospective nature of the study and the limited number of AMR cases are its chief drawbacks. On the other hand, this is the initial investigation of a relatively extensive and distinctive pediatric patient populations. The value of the study is derived from the comprehensive analysis of morphological changes in specific types of rejection. Morphological lesions are the least specific manifestation suggesting AMR, as demonstrated by an innovative, easy-to-use scale of histopathological grading scale of AMR (pAMR). The likelihood of an AMR diagnosis is statistically increased by a positive C4d staining, regardless of whether or not there are accompanying microscopic abnormalities.

## 5. Conclusions

The diagnosis of AMR in a transplanted liver is intricate and requires a multifaceted approach. However, the proposed histopathological grading may be a helpful method for selecting patients who should be assessed for donor-specific antibodies (DSAs) or in whom AMR should be suspected when DSAs cannot be determined.

## Figures and Tables

**Table 1:** Clinical characteristics of the patients.

Parameter	AMR	TCMR
Age (years)	median 2.99 (0.68–11.41)	median 2.77 (0.45–16.86)
Body weight (kg)	median 14.20 (8.20–33.50)	median 12.50 (6.0–65.0)
WBC (G/L)	median 7.37 (4.03–10.67)	median 7.03 (3.88–13.78)
Erythrocytes (mln/µL)	median 4.86 (3.82–4.98)	median 4.80 (2.27–5.59)
HGB (g/dL)	median 12.90 (9.30–14.40)	median 11.90 (7.80–15.10)
Bilirubin (mg/dL)	median 0.50 (0.40–1.60)	median 0.50 (0.20–42.18)
AST (U/L)	median 39 (23.0–49.0)	median 33 (16.0–224.0)
AlAT (U/L)	median 43 (13.0–60.0)	median 28.50 (10.0–125.0)
GGTP (U/L)	median 34 (11.0–350.0)	median 25 (10.0–998.0)
Creatinine (mg/dL)	median 0.50 (0.26–0.59)	median 0.47 (0.16–1.69)
Cystatin (mg/dL)	median 0.82 (0.78–1.20)	median 0.94 (0.74–8.89)
INR	median 1.06 (0.92–2.33)	median 1.09 (0.88–2.99)

WBC—white blood cells; HGB—hemoglobin; AST—aspartate aminotransferase; AlAT—alanine aminotransferase; GGTP—gamma-glutamyl transpeptidase; INR—International Normalized Ratio.

**Table 2:** The characteristics of patients with AMR.

Case Number	pAMR Grade	C4d Score	DSA Specificity with MFI Values	Key Histopathological Findings	Specific Treatment Administered	Treatment Response	Follow-Up Outcome
Patient 1	Two in all three biopsies	3	Class II anti-HLA (DP19) MFI 1065	Microvasculitis, portal capillaritis	Plasmapheresis, methylprednisolone, and IVIG	Improvement in clinical condition	Stable condition/no evidence of rejection or substantial fibrosis in the most recent protocol biopsy.
Patient 2	1I(+)	2	Class II anti-HLA (DQ2) MFI 3810	Microvasculitis, dilation of capillaries	ATG in conjunction with plasmapheresis	Improvement in clinical condition	Stable condition/minor nonspecific inflammation, no evidence of substantial fibrosis in the most recent protocol biopsy.
Patient 3	1I(+) first biopsy 2 second biopsy	3 2		Absence Microvasculitis, portal capillaritis, centrilobular/perivenular hepatocyte swelling, dilation of capillaries	IVIG and subsequently rituximab		Stable condition/no evidence of rejection or substantial fibrosis in the most recent protocol biopsy.
Patient 4	2	1	Class II anti-HLA (CD4) MFI 1065	Microvasculitis, portal capillaritis, centrilobular/perivenular hepatocyte swelling	IVIG with plasmapheresis	Improvement in clinical condition	PTLD (Classical Hodgkin’s Lymphoma) four years after an episode of rejection.
Patient 5	1I(+)	1	Class II anti-HLA (DP1) MFI 1692 MFI 1125	Absence	ATG	Improvement in clinical condition	Stable condition/minor nonspecific inflammation, no evidence of substantial fibrosis in the most recent protocol biopsy.
Patient 6	1I(+)	1	Class II anti-HLA (DQ2) MFO 1103	Absence	ATG	Improvement in clinical condition	Minor nonspecific inflammatory infiltrates, minimal steatosis, no evidence of substantial fibrosis in the most recent protocol biopsy.
Patient 7	2	1	Class I anti-HLA (A25) MFI 3247	Microvasculitis, portal capillaritis, centrilobular/perivenular hepatocyte swelling	IVIG	Improvement in clinical condition	Minor nonspecific inflammatory infiltrates, minimal steatosis, no evidence of substantial fibrosis in the most recent protocol biopsy.

IVIG—intravenous immunoglobulins; ATG—antithymocyte globulin; PTLD—post-transplant lymphoproliferative disease.

**Table 3:** Difference in liver parameters between AMR and ACR.

	AMR (*n* = 10)	ACR (*n* = 52)	*p*-Value
Bilirubin; median (Q1–Q3)	4.34 (1.64–8.59)	2.43 (0.79–5.62)	0.224
AST; median (Q1–Q3)	143.5 (96–206)	95 (78–156)	0.108
ALT; median (Q1–Q3)	224 (139–361)	179 (115–259)	0.368
GGTP; median (Q1–Q3)	340 (220–999)	309.5 (131–494.5)	0.438

U Mann–Whitney Test; AST—aspartate aminotransferase; GGTP—gamma-glutamyl transpeptidase.

## Data Availability

The original contributions presented in this study are included in the article. Further inquiries can be directed to the corresponding author.

## References

[1] Ayu P., Sebba A.K. (2021). Quality of life in patients after liver transplantation: A literature of review. Ann. Hepato-Biliary-Pancreat. Surg..

[2] Jadlowiec C.C., Morgan P.E., Nehra A.K., Hathcock M.A., Kremers W.K., Heimbach J.K., Wiesner R.H., Taner T. (2019). Not All Cellular Rejections Are the Same: Differences in Early and Late Hepatic Allograft Rejection. Liver Transplant..

[3] European Association for the Study of the Liver (2024). EASL Clinical Practice Guidelines on liver transplantation. J. Hepatol..

[4] Taner T., Stegall M.D., Heimbach J.K. (2014). Antibody-Mediated Rejection in Liver Transplantation: Current Controversies and Future Directions. Liver Transpl..

[5] Demetris A.J., Bellamy C., Hübscher S.G., O’Leary J., Randhawa P.S., Feng S., Neil D., Colvin R.B., McCaughan G., Fung J.J. (2016). 2016 Comprehensive Update of the Banff Working Group on Liver Allograft Pathology: Introduction of Antibody-Mediated Rejection. Am. J. Transplant..

[6] Lee B.T., Fiel M.I., Schiano T.D. (2021). Antibody-mediated rejection of the liver allograft: An update and a clinico-pathological perspective. J. Hepatol..

[7] Filippone E.J., Farber J.L. (2021). Histologic Antibody-mediated Kidney Allograft Rejection in the Absence of Donor-specific HLA Antibodies. Transplantation.

[8] Wu L.-N., Liu J.-Y., Zhao X.-Y., Zhu Z.-J., Wei L., Qu W., Zeng Z.-G., Sun L.-Y. (2025). Clinical Features and Treatment of Antibody-Mediated Rejection After Liver Transplantation: A Largest Single Center Experience in China. Clin. Transplant..

[9] Haas M. (2013). Pathology of C4d-negative antibody-mediated rejection in renal allografts. Curr. Opin. Organ Transplant..

[10] Berry G.J., Burke M.M., Andersen C., Bruneval P., Fedrigo M., Fishbein M.C., Goddard M., Hammond E.H., Leone O., Marboe C. (2013). The 2013 International Society for Heart and Lung Transplantation Working Formulation for the standardization of nomenclature in the pathologic diagnosis of antibody-mediated rejection in heart transplantation. J. Heart Lung Transplant..

[11] Orandi B.J., Alachkar N., Kraus E.S., Naqvi F., Lonze B.E., Lees L., Van Arendonk K.J., Wickliffe C., Bagnasco S.M., Zachary A.A. (2016). Presentation and Outcomes of C4d-Negative Antibody-Mediated Rejection After Kidney Transplantation. Am. J. Transplant..

[12] Nankivell B.J., P’Ng C.H., Shingde M. (2022). Glomerular C4d Immunoperoxidase in Chronic Antibody-Mediated Rejection and Transplant Glomerulopathy. Kidney Int. Rep..

[13] Sutanto H., Maimunah U., Fetarayani D. (2024). Donor-specific antibodies and their impact on antibody-mediated rejection post-liver transplantation: A comprehensive review. J. Liver Transplant..

[14] O’Leary J.G., Michelle Shiller S., Bellamy C., Nalesnik M.A., Kaneku H., Jennings L.W., Isse K., Terasaki P.I., Klintmalm G.B., Demetris A.J. (2014). Acute liver allograft antibody-mediated rejection: An inter-institutional study of significant histopathological features. Liver Transplant..

[15] Cicalese L., Walton Z.C., Du X., Kulkarni R., Qiu S., El Hag M., Stevenson H.L. (2024). Antibody-Mediated Rejection in Liver Transplantation: Immuno-Pathological Characteristics and Long-Term Follow-Up. Transpl. Int..

[16] Nocito A., El-Badry A.M., Clavien P.A. (2006). When is steatosis too much for transplantation?. J. Hepatol..

[17] Dixon W., Perito E.R., Feng S. (2022). Baby Steps: Understanding Allograft Fibrosis in Pediatric Liver Transplantation. Liver Transplant..

